# Vaccine Hesitancy during the Coronavirus Pandemic—Lessons from Polio

**DOI:** 10.3390/life11111207

**Published:** 2021-11-09

**Authors:** Lakshini Gunasekera, Tissa Wijeratne

**Affiliations:** 1 Department of Medicine and Neurology, Australia Institute of Muscularskelatal Sciences, Melbourne Medical School, St Albans, VIC 3021, Australia; lakshini.gunasekera@wh.org.au; 2 Department of Medicine and Neurology, Sunshine Hospital, St Albans, VIC 3021, Australia

The COVID-19 pandemic continues to cause disruptions to families, businesses and healthcare systems globally. As government-mandated lockdowns continue to come and go, the question is raised when we may start to gain more control of the situation and return to normal life. The rapid approval of several vaccines to tackle COVID-19 has been very timely and is a testament to the successful efforts of scientists worldwide. With this success, however, comes skepticism from the general public about their safety and utility, especially due to recent media reports of thrombotic complications such as cerebral venous sinus thrombosis (CVST) [1,2,3,4,5]. In this context, could mass vaccination be a way out of recurrent lockdowns and return to normal life? It is useful to reflect on poliomyelitis as an example of a similar crippling infectious disease that has largely been eradicated due to successful mass vaccination. There are many useful lessons to be learned from the poliomyelitis pandemic. In the following opinion piece, we explore similarities in management between these two viral infections, which are of interest from a public health perspective. 

There are many useful lessons to be learned from the poliomyelitis pandemic and its subsequent management on a global scale. It is clear that reducing morbidity and mortality is not dependent on advanced science, knowledge of virology or vaccine development. The science is here. The vaccines are here. We need equitable delivery of these vaccines around the world combined with mass vaccination in order to control this public health problem on a global scale. 

Many would consider poliomyelitis a disease of the past. Global cases have declined from 350,000 in 1988 across 125 countries, to a mere 33 cases in 2018 in two countries due to mass vaccination [6,7]. The Global Polio Eradication Initiative (GPEI) was formed with the main goal of ensuring no child ever experiences paralytic polio again, “reaching every last child” [8]. Due to the successful vaccination efforts of GPEI on a global scale, 18 million cases of paralytic polio have been avoided [8]. The work of the GPEI is literally life-changing. It aims to detect and halt all cases of polio transmission, strengthen local immunization programs and contain any outbreaks that occur [8]. We are at a revolutionary point in time when wild polio is close to being eradicated. Given the similarities between poliomyelitis and coronavirus, perhaps mass vaccination could be considered. 

The poliovirus and SARS Coronavirus-2 are viruses that enter our body via fecal–oral and respiratory routes, respectively. Asymptomatic cases are a major source of transmission for both conditions, and fortunately, most do not end with severe long-term consequences. Management of coronavirus so far has been similar to poliomyelitis management in the 1940s. For both, avoidance of large social gatherings, lockdowns of cities, closure of public places, homeschooling of children and mandatory masks have been used in the absence of an effective cure. The mental health and economic impact of both conditions, especially given the recurrent lockdowns, cannot be underestimated. What makes coronavirus more difficult to control than polio is the change in our lifestyles—recent high-speed air travel between different countries and globalisation of the economy makes it harder to contain infections. By the same token, however, we are living in a period of unprecedented technological growth with information creation, access and sharing on a global scale. Perhaps we can use the latter to our advantage and harness this power to ensure fair resource allocation and distribution.

Medicine has come a long way from paternalistic care to a patient-centered approach where vaccination is seen as an individual decision. However, has the pendulum swung too far away from healthcare providers to be able to protect patients? We see a rise in polio cases as vaccination rates decline in endemic areas. Could mass coronavirus vaccination avoid a similar fate?

Concerns about vaccine safety are not new. In 2019, the World Health Organisation cited vaccine hesitancy to be a global threat [9]. Refusal of COVID-19 vaccination reduces the likelihood that we will achieve herd immunity and therefoe, reduction or elimination of this virus from the community. The safety of these rapidly approved vaccines continues to be a limitation in their widespread delivery even amongst healthcare workers [10]. Of course, these vaccines go through multiple phases of laboratory and clinical testing involving tens of thousands of volunteers prior to their approval by independent federal bodies [11]. A contributor to this hesitancy is distrust in the messenger RNA (mRNA) vaccine backbone, even though this has been used in vaccines for over 30 years with progressive improvements to its structure [12]. Indeed, two of the most popular vaccine rollouts from Pfizer and Moderna both use this mRNA backbone, and its rapid availability commercially was dependent on this technology having been available for them to build on [13]. Studies also show that cerebral venous sinus thrombosis is only rarely associated with COVID-19 vaccines and does not appear to be causative in the majority of patients and thus should not be a reason to dissuade vaccination [14]. Perhaps if more education for the public were made available about the development of these vaccines using previously available scientific concepts and the phases of vaccine testing for safety, there may be more public acceptance of vaccination.

We know that immunity can be developed either through natural infection with the virus or vaccination [15]. Some individuals express a preference to naturally be infected with the coronavirus to develop antibodies rather than through vaccination, as they believe the coronavirus pandemic to be a sensationalist story created by the media [15]. There is evidence that some individuals think the risk of vaccination is higher than the risk of morbidity and mortality from coronavirus infection itself [15]. This is quite contrary to the evidence that over 2 million individuals have died from coronavirus since it was first discovered [16]. Distrust in the government may play a part in this high death rate [5], though it is unclear how this can be reversed acutely. Patients may have more trust in their physicians rather than politicians [17,18], so perhaps more doctors should be leading this public health crisis. 

We have seen poliomyelitis largely being eradicated from the world due to consistent vaccination efforts for over 60 years [8]. It is worthwhile to consider whether mass vaccination for coronavirus may help end recurrent lockdowns. Reasons for vaccine hesitancy cited above may be alleviated by more physicians leading this healthcare crisis by delivering health information and education directly to the public rather than through politicians. Perhaps this may allow more confidence, enhanced uptake of vaccination and therefore better herd immunity.

## Data Availability

Not applicable.
